# Supplementary material for: The Role of Research in Guiding Treatment for Women’s Health: A Qualitative Study of Traditional Chinese Medicine Acupuncturists
Source: Int J Environ Res Public Health. 2021 Jan 19;18(2):834. doi: 10.3390/ijerph18020834 (PMC7835913; doi:10.3390/ijerph18020834)
Supplement: Supplementary file 1 [file ijerph-18-00834-s001.pdf]

Text S1. Interview guide:

1. Can you briefly tell us your name, what part of the country you practice in and how many years you have been in practice ?
2. How do most of these women end up at your clinic (explore referral patterns) ?
3. Do you communicate with the women's other health-care providers?
4. When women come in to see you, how do you design your treatment plan ? (explore sources of knowledge here as well)
5. What frequency of treatment do you normally use ? are there any barriers to this ?
6. What do you do to keep your knowledge of women's health up to date ?
7. How valuable are academic articles such as randomised controlled trials for your practice ? Do they influence your practice at all ? Why/ Why not?
8. Is there anything that would help make research results easier to translate into your clinical practice ?
